# Patchy Distribution of Potato Cyst Nematodes Within Single Arable Fields Reveals Local Disease Suppressiveness Mediated by Disparate Microbial Communities

**DOI:** 10.1111/1462-2920.70113

**Published:** 2025-05-26

**Authors:** Robbert van Himbeeck, Stefan Geisen, Casper van Schaik, Sven van den Elsen, Roeland Berendsen, André Bertran, Egbert Schepel, Johannes Helder

**Affiliations:** ^1^ Laboratory of Nematology Wageningen University Wageningen the Netherlands; ^2^ Plant–Microbe Interactions, Institute of Environmental Biology, Department of Biology, Science4Life Utrecht University Utrecht the Netherlands; ^3^ HLB BV Wijster the Netherlands

**Keywords:** disease suppressive soils, environmental microbe–pathogen interactions, microbial antagonists, plant–parasitic nematodes, soil microbial communities

## Abstract

Disease suppressiveness is a complex phenomenon that is assumed to be the resultant of the actions of local microbial antagonists. Exploitation of disease suppressiveness as a tool to manage pathogens is hindered by our poor understanding of this phenomenon. Here we investigated soil microbiome‐based suppression of potato cyst nematodes (PCN), and to this end, four apparently homogeneous potato fields with an unexplained non‐homogeneous PCN distribution were selected. We hypothesised that this patchy PCN distribution resulted from local variation in disease suppressiveness. Under controlled greenhouse conditions, we confirmed the overall suppressiveness of these soils vis‐à‐vis PCN and soils were gamma‐irradiated to corroborate the biotic origin of this suppression. Subsequent DNA‐based analysis of the microbial community in the potato rhizosphere revealed suppressiveness‐related contrasts in community composition between suppressive and conducive patches. Elevated abundances of fungal (e.g., *Metacordyceps chlamydosporia*) and bacterial (e.g., *Pseudomonas fluorescens* ) nematode antagonists were positively associated with PCN suppressive patches. Distinct sets of antagonists were found to be associated with PCN suppression despite the geographical closeness of the locations under investigation. Our findings confirm the biotic origin of local PCN suppressiveness and reveal that disparate microbial communities could achieve similar outcomes.

## Graphical abstract

We investigated whether a patchy field distribution of potato cyst nematodes (PCN) resulted from local heterogeneity in disease suppression. The biotic origin of suppression in soils was confirmed, and elevated abundances of fungal (e.g., *Metacordyceps chlamydosporia*) and bacterial (e.g., *Pseudomonas fluorescens* ) nematode antagonists were positively associated with PCN suppressive patches.

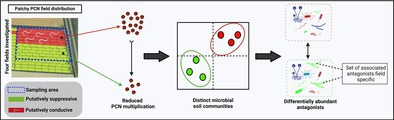

## 1 Introduction

To restore and protect natural and arable soils, numerous agrochemicals that used to be applied for plant–pathogen control are phased out in many global regions (e.g., in the European Union (2022)). Crop farmers consequently have an increasingly narrow set of control measures at their disposal and especially for soil‐borne diseases, novel management strategies are needed. The exploitation of local disease‐suppressive potential of the soil microbiome is a promising approach that could contribute to plant–pathogen control (e.g., Expósito et al. 2017). The first description of local disease suppression in soil dates back nearly a century (Henry 1931), and since that time, disease‐suppressive soils have been scrutinised to isolate and characterise microbial antagonists responsible for this phenomenon. Decades of research have resulted in the discovery of a wide range of natural enemies of plant–pathogenic bacteria (Abo‐Elyousr and Hassan 2021), fungi (Penton et al. 2014; Bubici et al. 2019), and nematodes (Li et al. 2015; Topalović et al. 2020). Based on these findings, numerous biological control agents have been developed (e.g., Li et al. 2015; Bubici et al. 2019), but in general, non‐native microorganisms show difficulties in establishing in local, highly competitive soil microbial communities. Local microbes have ample competitive competences within their native soil ecosystem. Although it has been shown that stimulating the local pathogen‐antagonistic community is feasible, for example through the use of cover crops (Cazzaniga et al. 2023, 2025), our limited understanding of the underlying biological processes (Expósito et al. 2017) currently hampers the broader adoption of this approach as a pathogen management strategy. Characterisation and ecological understanding of the microbiome of suppressive soils are essential in harnessing its potential in management strategies. The comparison of microbiomes from suppressive and conducive fields can be complicated if those fields are spatially dispersed, have disparate soil types, or are managed differently. Ideally, one would like to study visually homogeneous fields with an unexplained patchy distribution of a given soil‐borne pathogen, while cropping and disease history suggests this pathogen should have been present throughout the field. Such a patchy pathogen distribution could result from hard‐to‐observe local variations in abiotic conditions or from the recent introduction of the pathogen. However, the success of pathogen invasion might also be caused by local heterogeneity in the composition and functioning of soil microbiomes (Wei et al. 2019; Gu et al. 2022). High‐resolution data on the within‐field distribution of soil‐borne pathogens would be a good starting point for investigating the nature of local disease suppressiveness, but such data are scarce. In this respect, soil‐borne pathogens with quarantine status might form an exception, as statutory regulations prompt farmers to have their fields sampled on a regular basis and with a relatively high sampling intensity.

Potato cyst nematode (PCN) is the common name for two quarantine pathogens, *Globodera pallida* and *G. rostochiensis*, that are present in virtually all major potato growing regions in the world. These pathogens constitute a major threat to global potato production, causing an estimated global yield loss of 9% (Jones et al. 2013). Because of its quarantine status, and given that both seed and consumer potatoes are large export commodities, this soil‐borne pathogen is carefully monitored both by plant health authorities and producers. As a result, the distribution of PCN is well‐known, and numerous distribution maps are available. Although most fields used for potato production are labelled as either non‐infested or infested with PCN, high‐resolution screenings occasionally reveal a patchy within‐field PCN distribution. Particularly, these fields are suitable objects to study local suppressiveness vis‐à‐vis this pathogen. PCN suppressive soils and antagonists have been documented in North‐Western Europe (Jones 2024; Cronin et al. 1997; Velvis and Kamp 1996), demonstrating the capacity of the region's soil microbiome to suppress this pathogen. However, PCN suppressive soils are considered to be uncommon (Kerry et al. 2009) and the local soil microbiome of PCN suppressive fields is understudied. Comparison of microbiomes associated with conducive and suppressive patches within single fields might allow us to pinpoint how to manipulate and control suppressive soils.

The aim of this study was to identify within‐field differences of PCN suppression and to characterise the associated microbial soil communities. We selected four apparently homogeneous arable fields that showed clear within‐field differences in PCN density. We verified whether these patchy PCN distributions had a biotic origin and—upon showing the biotic origin—we investigated whether the virtual absence of PCN was associated with the presence of nematode antagonists. High‐throughput DNA sequencing was used to characterise fungal and bacterial rhizosphere communities and to identify potential nematode antagonists. With this experimental setup, we addressed the following research questions: (1a) Can the apparently conducive and suppressive nature of patches within the selected potato production fields be reproduced under greenhouse conditions? (1b) Do observed within‐field differences in PCN infection have a biotic origin?; (2) What assemblages of antagonists are associated with PCN suppression; and (3) how do these assemblages vary among suppressive patches between fields?

## 2 Methods and Materials

### 2.1 Field Selection and Sampling

**FIGURE 1:**
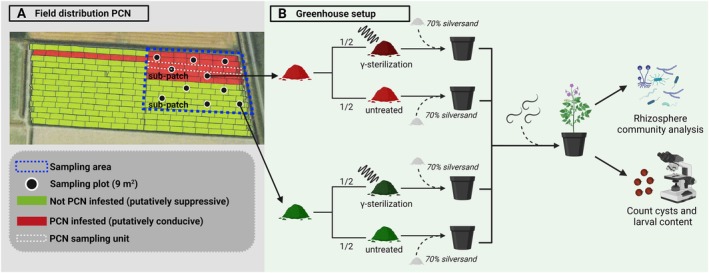
Overview of the experimental setup. (A) An example of PCN distribution data (based on PCN sampling units—white dashed line) and soil sampling scheme. Non‐PCN infested (i.e., putatively suppressive) and PCN infested (i.e., putatively conducive) sub‐patches similar in size were sampled within the sampling area (blue dashed line). In each sub‐patch, a composite sample was collected from five random sampling plots. (B) Of each sampling plot, one third of the collected soil was sterilised using γ‐irradiation, the remaining two thirds of soil were kept untreated. Field soil (30%) was then mixed with steam‐sterilised silversand (70%), and inoculated with 10,000 PCN eggs before planting a potato tuber. The un‐inoculated negative controls are not included in this figure. After 13 weeks, the potato rhizosphere was collected for microbial community analysis. After 16 weeks, PCN cysts were collected and quantified [Created with BioRender].

To study PCN suppressiveness, visually homogeneous fields with a patchy PCN distribution were identified (Figure 1A). For this, we mined a proprietary database generated by HLB BV (Wijster, the Netherlands), an agricultural consultancy company. This database comprises high‐resolution PCN maps of hundreds of arable fields collected over a range of years. For these maps, fields were sampled using an automated soil core collector taking samples every 2 m in rows with a width of 6 or 9 m, and up to 150 m in length. Each such row was used to determine an individual PCN density value, representing one ‘PCN sampling unit’ (Figure 1A, white dashed line). Upon microscopic analysis distribution maps of PCN of entire fields were generated with spatial resolution corresponding to the size of the individual PCN sampling units (see, e.g., Figure 1A). After consulting the farmers to verify field homogeneity, four PCN patchy‐fields were selected in Friesland (a northern province in the Netherlands): field E (farmer 1), G (farmer 2), K (farmer 2), and S (farmer 3). The two furthest apart fields were separated by 30 km. The infested and non‐infested patches of field K were separated by a narrow ditch. PCN distribution of selected fields was determined recently (< 3 years). Patches of the field that were infested with PCN were labelled ‘putatively conducive’ patches, while non‐infested are referred to as ‘putatively suppressive’ patches. Patchy fields were sampled at the end of March 2023 (before the main growing season). Within each field, the putatively suppressive and conducive patches were different in size. Hence, for each field a sampling area was defined that consisted of two adjacent, similar‐sized sub‐patches that were either putatively suppressive (i.e., not PCN infested) or conducive (i.e., PCN infested) (see, e.g., blue dashed line in Figure 1A). This resulted in sampling areas of 0.23, 1.35, 11.5, and 0.65 ha for, respectively, fields E, G, K, and S. For each sub‐patch, five random sampling squares of $9\;m^{2}$ were plotted in both the putatively conducive and suppressive sub‐patches, resulting in 10 sampling plots per field. For each plot, 14 soil cores were collected using an auger (ø4 cm, 0–25 cm deep, Royal Eijkelkamp). These subsamples were pooled, and soil was mixed thoroughly. Per field four samples of each 100 g (two from each of the putatively conducive and suppressive sub‐patches) per field were collected for chemical analyses (see Section 2.2). Putatively conducive sub‐patches were always sampled before putatively suppressive sub‐patches to prevent potential contamination with antagonistic microbial taxa. Between sub‐patches of a field, all materials were cleaned with 70% ethanol. The soil was transferred to the greenhouse at the day of sampling, and air‐dried at ambient temperature.

### 2.2 Chemical Analyses of Soil

For each sub‐patch (infested or non‐infested) of each field, 2 sampling plots were randomly selected and processed for chemical analysis. Approximately 100 g of the collected composite sample was dried overnight at 40°C. The soil samples were then crushed, and thereafter all samples were sieved using a 2 mm grid sieve. A total of 35 g of sieved soil was used for the determination of total N, total P, organic‐C, pH, and % organic matter at Farm Systems Ecology (Wageningen, the Netherlands).

### 2.3 Greenhouse Setup and Soil Sterilisation

Collected soil was air‐dried at ambient temperature for 2 weeks. Dried soil was then packed in sturdy plastic bags and crushed mechanically. One third of this crushed soil was sterilised by γ‐irradiation (> 30 kGy) (Synergy Health Ede B.V., the Netherlands). For each sampling plot, 2 L pots were filled with a mixture (based on volume) of 30% non‐sterilised soil or sterilised soil, and 70% steam‐sterilised silver sand (in quintuple). Nematode suppressiveness is—just like other types of disease suppressiveness—readily transferred and 30% field soil should suffice to induce suppressiveness in the pots in case a suppressive microbiome was present in the original field samples (Westphal and Becker 2000). Approximately 3 weeks before the start of the experiments, potato tubers (cultivar Desirée, fully susceptible to *G. pallida* ; Agrico B.V., the Netherlands) were thoroughly washed with tap water to remove any adhering particles, dried with a clean paper cloth, and subsequently allowed to sprout in the dark at ambient temperature. To prepare inoculum, *G. pallida* line D383 cysts were soaked in tap water for 24 h at room temperature and thereafter crushed using a glass rod. Egg density was determined microscopically. Before inoculation, the moisture content of each pot was brought to ±15%. Each pot was inoculated with 10,000 PCN eggs. Inoculation needles (30 mm length, ø4 mm, side opening 25 mm above the bottom end) were used to guarantee a proper vertical inoculum distribution in each pot as described in Teklu et al. (2018). In brief, after inserting the inoculation needles in the soil, the nematode egg suspension was pipetted in the needles (using a glass pipette tip) while simultaneously pulling the needles slowly upwards. Nematode eggs were delivered at five positions per pot. As a positive control for PCN infectiousness, five pots filled with an artificial soil mixture optimised for PCN multiplication were inoculated with PCN. This artificial soil mixture consisted of silver sand (70% wt/wt), hydro granules (18% wt/wt, Lecaton 2–5 mm), and kaolin clay powder (12% wt/wt), to which NPK (12:10:18) fertiliser granules (0.001% wt/wt) were added (Teklu et al. 2018). To check for the presence of any PCN in the sampled soil, a negative control (= no inoculation with PCN) was included for all plots. After inoculation, a potato tuber containing two sprouts was planted in each pot. The pots were distributed in the greenhouse in a randomised complete block design. The greenhouse conditions were as follows: 20°C (day)/15°C (night) including table cooling and ±70% relative humidity. The greenhouse was illuminated daily with artificial growing light from 06:30 to 09:30, after which natural sunlight provided illumination until sunset. To retain the soil moisture content around 15%, a subset of pots was weighted 3 times per week. Weight loss due to water evaporation was assessed while taking plant biomass accumulation into account, and water was added accordingly. Plants were regularly fertilised with Hyponex fertiliser (Royal Brinkman, the Netherlands).

### 2.4 Rhizosphere Sampling, PCN Cysts Harvesting, and Cyst Counting

After 13 weeks, rhizosphere samples were collected from all pots, thereby avoiding co‐collection of PCN cysts. Plants were carefully lifted from their pots, and transversally cut plastic pipette tips (opening ±5 mm) were used to scrape the soil from the roots. In total, approximately 5 g of rhizosphere soil was collected per pot. The rhizosphere soil was snap frozen in liquid nitrogen immediately after collection, kept on dry ice after collection, and subsequently stored at −80°C.

To ensure fully ripened PCN cysts, the potato plants and pots were allowed to dry for 3 weeks after rhizosphere collection. The cysts were harvested from all pots using the Seinhorst method (Seinhorst 1964) 16 weeks after inoculation. Extracted cysts were cleaned with a spray nozzle over a 900 μm sieve, and thereafter air‐dried at room temperature. The number of cysts and larval contents was microscopically quantified by HLB BV (Wijster, the Netherlands).

### 2.5 DNA Extraction and Amplification of the Bacterial 16S rDNA Gene

DNA was extracted from 2 g of rhizosphere soil according to Harkes et al. (2019). The DNA concentrations of all samples were determined using a Qubit (Thermo Fisher Scientific Inc.) and subsequently all DNA samples were diluted to 1 ng/μL. Nearly complete bacterial 16S rDNA was amplified using universal 27F‐CM (AGAGTTTGATCMTGGCTCAG) (Frank et al. 2008) and 1492R (CGGTTACCTTGTTACGACTT) primers that were tailed with barcode sequences of the EXP‐NBD196 kit (Oxford Nanopore Technologies plc., UK). PCR was performed in simplex, and each PCR reaction consisted of 12.5 μL Q5 High‐Fidelity 2× MasterMix (NEB), 200 nM of each primer (IDT), 7.5 μL of UltraPure DNase/RNase‐Free distilled water (Invitrogen) and 3 μL of DNA template (1 ng/μL). The following PCR scheme was used: initial denaturation at 98°C for 30 s, followed by 25 cycles of 98°C for 10 s, 58°C for 30 s, 72°C for 60 s, with a final extension step at 72°C for 2 min. After PCR, amplification success and amplicon fragment size were verified on a 1.5% agarose gel.

### 2.6 Nanopore (16S rDNA) and Illumina (ITS2) Sequencing

The 16S rDNA PCR products were merged (5 μL of each) in two pools and thereafter bead‐cleaned (0.75 bead:sample ratio) using NucleoMag NGS Clean‐up and Size Select beads to remove contaminants and small unwanted DNA fragments. In total 200 fmol of each pool was prepared for nanopore sequencing using the SQK‐LSK114 kit (Oxford Nanopore Technologies plc., UK) following the manufacturer's instructions. Libraries were then loaded on R10.4.1 flowcells (FLO‐MIN114). The sequencing was performed on a MinION Mk1C and raw reads were outputted in POD5 file format.

For the characterisation of the fungal community, DNA extracts were sent to Genome Québec (Montréal, Canada) for amplification of the ITS2 region (primers gITS7: GTGARTCATCGARTCTTTG and ITS4R: TCCTCCGCTTATTGATATGC), Illumina library preparation, and subsequent Illumina NextSeq (2 × 300 bp) sequencing.

### 2.7 Bio‐Informatic Analyses

The raw POD5 files resulting from the 16S nanopore sequencing were basecalled using the super‐accuracy basecalling model (v5) of Dorado (v0.7.2). The resulting .fastq files were demultiplexed using Dorado, and simultaneously ONT adapters and barcodes were removed. The reads were filtered on length (min. 1000 bp, max. 2000 bp) and quality (> Q20) using NanoFilt (v2.8.0; De Coster et al. 2018), before primer removal using cutadapt (v4.6; Martin 2011). The taxonomic identification of the resulting reads was performed with Emu (v.3.4.5; Curry et al. 2022), a taxonomic identifier specifically developed for Nanopore reads, using the Silva 16S rDNA database (release 138.1; Quast et al. 2012). Unlike OTU or ASV‐based analyses, Emu classifies individual reads and assigns them directly to a taxon, providing taxon‐level read counts. The resulting Emu files were subsequently merged using the parse_silva_output.py Python script from the CoatOfArms GitHub repository (https://github.com/gbouras13/coatofarms). This script was modified to also output the read count data.

For the fungal communities, demultiplexed .fastq files containing raw ITS2 reads were obtained from Genome Québec (Montréal, Canada) and these reads were further processed using QIIME2 (2024.2 amplicon distribution; Bolyen et al. 2019). The fungal ITS2 region was extracted from the reads using ITSxpress (v2.0.2; Rivers et al. 2018), and in this step also the primers were also removed from the reads. Next, DADA2 (v2024.2.0; Callahan et al. 2016) with no truncation, was used to de‐noise and merge the reads and to remove chimeric reads. The resulting ASVs were clustered at 97% similarity using VSEARCH (v2024.2.0; Rognes et al. 2016) as recommended for fungal ITS sequences (Kauserud 2023; Tedersoo and Anslan 2019). Next, the reads were taxonomically identified using the QIIME2 classify‐sklearn command of the feature‐classifier plugin and the pre‐trained UNITE (v10.0) database (Abarenkov et al. 2024).

### 2.8 Statistical Analyses

All analyses were performed in R (v4.3.1; RCoreTeam 2013). The overall difference in cyst quantities between the putatively suppressive and conducive sub‐patches (i.e., variable ‘sub‐patch’) was analysed using a Generalised Linear Model (GLM) of the MASS package (v7.3‐60; Venables and Ripley 2002). To compensate for the presence of cysts in the sampled field soil, the number of cysts in the non‐inoculated negative controls of each sampling plot was subtracted from those in the inoculated pots. Cyst quantitative data were analysed using a GLM with a Negative Binomial (NB) distribution. For the sterilised soil data, one extreme outlier (cyst count = 44, field G, suppressive sub‐patch) was removed as this prevented the use of GLM models and resulted in overdispersed models. The differences in number of living larvae per cyst were also analysed with GLM‐NB models. All models with an NB distribution showed no over‐ or underdispersion (tested with the performance package v0.13.0; Lüdecke et al. 2021), except for the comparison of the number of living larvae for field K. For the latter analysis, a Poisson regression was used due to equidispersion of the data. Violin plots were created to visualise the cyst quantity data using ggplot2 (v3.4.2; Wickham 2016).

The QIIME2 OTU table and taxonomy file of the fungal ITS2 reads were imported into R phyloseq objects using the qiime2R package (v0.99.6; Bisanz 2018). Fungal OTUs with < 10 reads were removed from the feature table. The total fungal OTU richness and mean richness for the putatively conducive and suppressive parts were determined with phyloseq (v1.42.0; McMurdie and Holmes 2013), after rarefying the read data to the lowest read count (16,438 reads). The mean richness data was checked for normality using Shapiro–Wilk tests and subsequently Student's *t*‐tests were used to compare the mean richness per field between the putatively conducive and suppressive parts. To visualise the community differences between the infested and uninfested sub‐patches, PCoA plots were created for all fields combined and each field separately using Bray–Curtis (Bray and Curtis 1957) and robust Aitchison (Gloor et al. 2017; Martino et al. 2019) dissimilarity matrices. For the Bray–Curtis dissimilarity matrices, the rarefied read data was used. For the robust Aitchison distance, the OTU table was first rclr transformed using QsRutils (v0.1.5; Quensen 2020) and thereafter an Euclidean distance was applied when performing the ordination (Gloor et al. 2017). Subsequently, PERMANOVA tests (adonis2, vegan package v. 2.6‐4; Oksanen et al. 2022) with 1000 permutations were used to statistically determine the effect of variables block, field, and sub‐patch (= putatively conducive or putatively suppressive) on the fungal communities, using both the Bray–Curtis and robust Aitchison distance. Differentially abundant taxa between the sub‐patches within each field were determined by using ANCOM‐BC with structural zeros detection (v1.4.0; Lin and Peddada 2020) and DESeq2 (v1.40.2; Love et al. 2014) with the ‘poscount’ size factor estimator to account for features with zero counts. The significance threshold of the Holm (ANCOM‐BC) and Benjimani–Hochberg (DESeq2) adjusted *p* values was set at 0.05. For improved visualisation, the DESeq2 log2 fold changes where shrank using the ‘apeglm’ estimator. The differential abundance results were visualised by creating a heatmap using the ComplexHeatmap package (v2.16.0; Gu 2022). Only putatively fungal taxa that are known to harbour nematode antagonists are shown. The taxa table resulting from the bacterial 16S rDNA sequencing was analysed using the same methods as those applied to the fungal ITS2 OTU table, with the following differences: a minimum abundance threshold of 0.0001 was used to retain taxa, and a rarefaction depth of 110,000 reads was used for Bray–Curtis dissimilarity. The bacterial mean richness data of all fields aggregated was not normally distributed, and therefore a Wilcoxon rank‐sum test was used in that instance.

For the identification of putatively nematode antagonists, we listed bacterial and fungal genera that were included in two reviews, namely Topalović et al. (2020) and Li et al. (2015), and a book by Stirling (2014). It is noted that some genera are dominated by nematode antagonists (e.g., the bacterial genus *Pasteuria*), while for other genera nematode antagonists constitute a small minority only (e.g., the bacterial genus *Pseudomonas*). This list includes genera with representatives that directly parasitize plant–parasitic nematodes (PPNs), as well as microbial taxa that are known to promote PAMP‐triggered immunity in their host plant.

## 3 Results

### 3.1 Verification of PCN Suppressiveness Under Controlled Conditions

**FIGURE 2:**
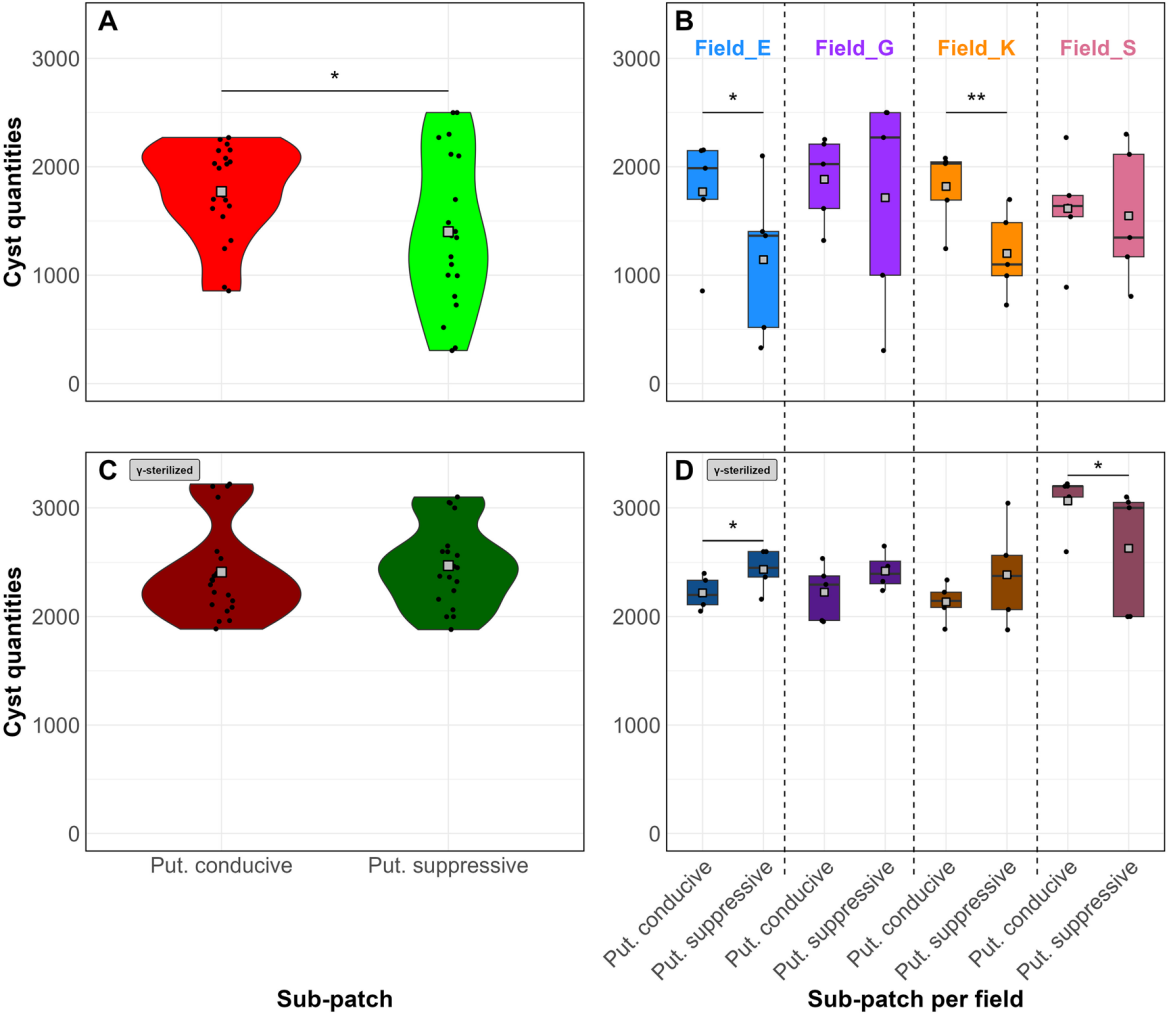
Number of potato cyst nematode (PCN) cysts per pot quantified at 16 weeks post‐inoculation on fully susceptible potato plants. (A) Cyst counts upon exposure to soil from putatively suppressive (green) and conducive (red) sub‐patches from the four different fields together (*n* = 20) and (B) PCN multiplication per field (*n* = 5). (C, D) PCN multiplication upon exposure to γ‐sterilised soil from putatively conducive (dark red) and suppressive (dark green) sub‐patches across all fields (*n* = 20) (C) and per field (*n* = 5) (D). *p < 0.05 and **p < 0.01 indicate statistical significant difference as determined by a GLM with a Negative Binomial distribution. Put., putatively.

To see whether apparent PCN suppressiveness under field conditions could be reproduced under controlled greenhouse conditions, PCN multiplication on sterilised and non‐sterilised soil from putatively conducive and suppressive sub‐patches was compared. The overall number of cysts in the non‐sterilised soils, quantified at the end of the experiment, was 21% lower (GLM.NB, *p* = 0.042) in the putatively suppressive sub‐patches of the field than in the conducive sub‐patches (Figure 2A). A subsequent per field comparison of PCN multiplication showed that for field E (GLM.NB, *p* = 0.025) and Field K (GLM.NB, *p* = 0.003) the number of cysts quantified in the putatively suppressive sub‐patch was, respectively, 35% and 34% lower than in the conducive sub‐patch (Figure 2B). For field G (GLM.NB, *p* = 0.17) and field S (GLM.NB, *p* = 0.53), there were no significant differences in the average number of cysts between the sub‐patches. When the soil was γ‐sterilised, the observed overall difference in PCN multiplication between the putatively conducive and suppressive soil was no longer present (GLM.NB, *p* = 0.42) (Figure 2C). Field K (GLM.NB, *p* = 0.097) showed no differences in cyst quantities between putatively suppressive and conducive sub‐patches anymore after γ‐sterilisation, and in field E the reduction in PCN multiplication in the putatively suppressive soil was also nullified (Figure 2D). Therefore, we conclude that the observed suppressiveness was caused by biota present in these soils. For field S, the number of cysts in the sterilised putatively suppressive soil was 14% lower (GLM.NB, *p* = 0.03) as compared with soil from the putatively conducive sub‐patch. This suggests that heterogeneous edaphic factors in Field S might have contributed to the observed reduction in PCN manifestation. For field G, no differences (GLM.NB, *p* = 0.06) in cyst quantities between putatively suppressive and conducive soil were observed after sterilisation.

Sterilisation of the putatively suppressive soil increased PCN multiplication in fields E (113%, GLM.NB, *p* < 0.00001), K (99%, GLM.NB, *p* < 0.00001), and S (70%, GLM.NB, *p* = 0.0003) (Figure S1B). For field G, sterilisation increased the number of PCN cysts by 41%, but this change was not significant (GLM.NB, *p* = 0.075). Although to a lesser extent, sterilisation also increased the number of PCN cysts on potato plants exposed to conducive soil (Figure S1D). Thus, γ‐sterilisation had a larger impact on PCN multiplication in soil from putatively suppressive sub‐patches than in soil from conducive sub‐patches.

Next to the number of cysts, the overall number of living larvae per cyst was counted. This parameter did not significantly differ between soil from putatively conducive and suppressive patches (GLM.NB, *p* = 0.58, Figure S2A). Field K was exceptional as cysts from pots with putatively suppressive soil comprised 8.5% (GLM.NB, *p* = 0.049) fewer living larvae as compared with conducive soil (Figure S2B).

### 3.2 Conducive and Suppressive Field Sub‐Patches Show Contrasting Fungal and Bacterial Communities

The complete bacterial 16S rDNA and fungal ITS2 region were sequenced to analyse the microbial rhizosphere communities (mean reads per sample respectively 167,455 and 79,191). After filtering, we identified a total of 1431 bacterial and 560 fungal taxa for all fields (Table 1). Per field, a total of 174–341 fungal and 865–1193 bacterial taxa were identified (Table 1). No significant differences in mean fungal OTU richness were observed between the putatively conducive and suppressive sub‐patches. The mean bacterial richness was higher for the putatively suppressive sub‐patches when the data from the four locations were aggregated (10.4% increase, Student's *t*‐test, *p* = 0.016). With a richness increase of 39.2% (Wilcoxon rank‐sum test, *p* = 0.009) as compared with the putatively conducive sub‐patch, field E was exceptional.

**TABLE 1:** Number of fungal OTUs and bacterial taxa (see Section 2.7) after filtering low abundant taxa.

	Fungal OTUs			Bacterial taxa		
		Mean no. of OTUs			Mean no. of taxa	
	Total no. of OTUs	Put. con	Put. sup	Total no. of taxa	Put. con	Put. sup
Fields aggregated	557	57^a^	64^a^	1431	550^a^	607^b^
Field E	337	76^a^	92^a^	1193	483^a^	672^b^
Field G	203	42^a^	68^a^	874	568	591^a^
Field K	172	49^a^	37^a^	875	592^a^	594^a^
Field S	212	59^a^	61^a^	865	557^a^	571^a^

*Note:* Total number of OTUs and taxa are given for all fields aggregated and for each field separately. Mean OTU and taxa numbers are presented for the putatively conducive and suppressive field sub‐patches. Letters are used to indicate significant differences between the mean OTUs and taxa per field (i.e., row‐wise per taxonomic group), according to the Student *t*‐test (*p* < 0.05).

**FIGURE 3:**
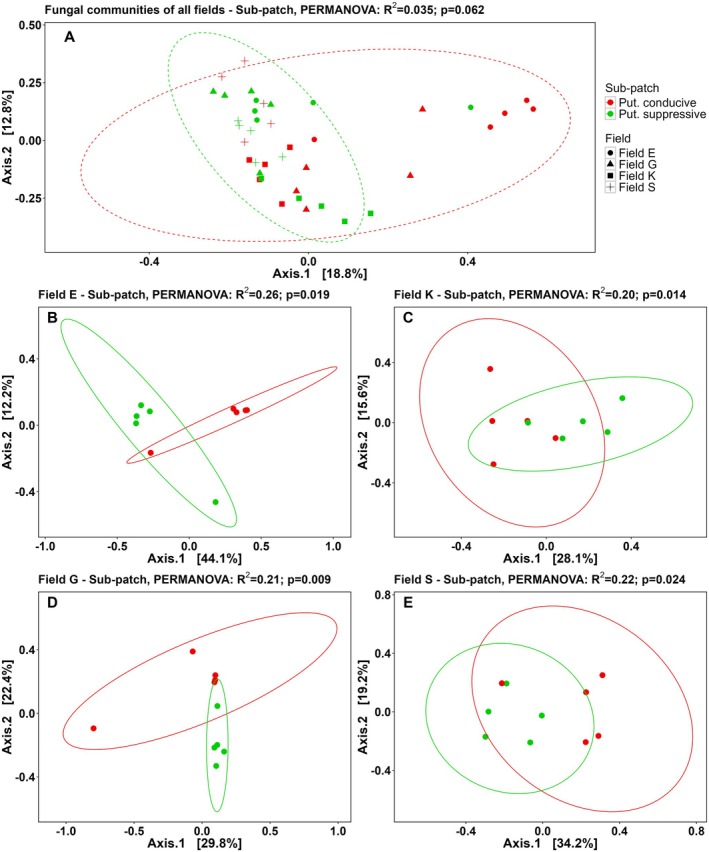
PCoA plots based on the Bray–Curtis dissimilarity matrix of fungal communities in rhizosphere samples collected from potato plants 13 weeks after inoculation with potato cyst nematodes. (A) Fungal communities in rhizosphere of plants exposed to soil from putatively conducive (red) or suppressive (green) sub‐patches from all four field locations and (B–E) at individual field location level (*n* = 5). B = field E, C = field K, D = field G, and E = field S. Next to (A), symbols for the individual field locations are presented. Figure headers include the PERMANOVA result of factor ‘sub‐patch’ (= putatively suppressive or conducive). Put., putatively.

**FIGURE 4:**
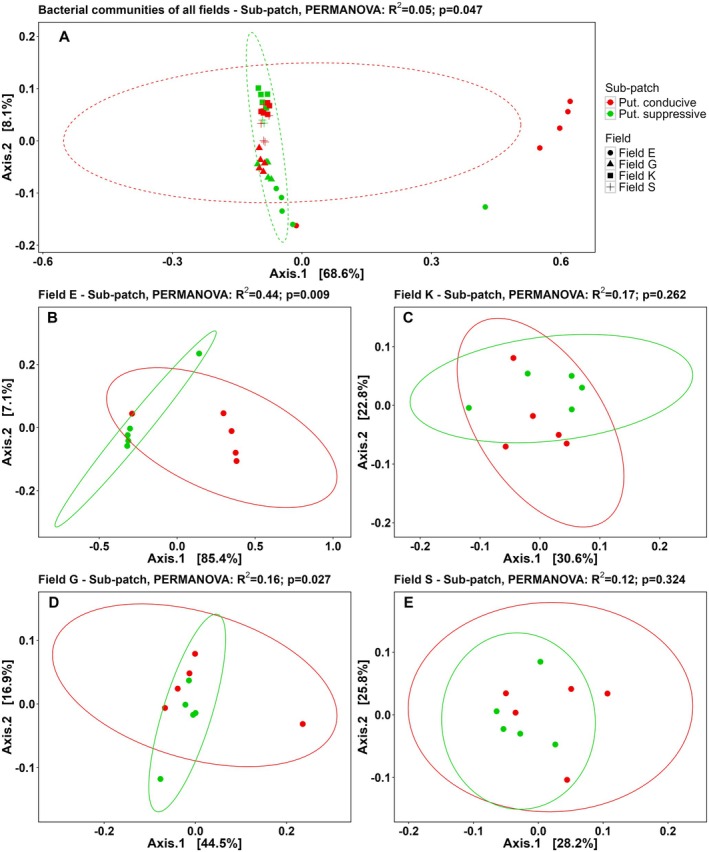
PCoA plots based on the Bray–Curtis dissimilarity matrix of the bacterial communities in rhizosphere samples collected from potato plants 13 weeks after inoculation with potato cyst nematodes. (A) Bacterial communities in rhizosphere of plants exposed to soil from putatively conducive (red) or suppressive (green) sub‐patches from all four field locations, and (B–E) at individual field location level (*n* = 5). B = field E, C = field K, D = field G, and E = field S. Next to (A), symbols for the individual field locations are presented. Figure headers include the PERMANOVA result of factor ‘sub‐patch’ (= putatively suppressive or conducive). Put., putatively.

PCoA plots (Bray–Curtis dissimilarity) were generated to visualise differences in the overall fungal (Figure 3) and bacterial (Figure 4) communities between the putatively conducive and putatively suppressive field sub‐patches (i.e., variable ‘sub‐patch’) of all fields under investigation. In general, the fungal communities of each of the four fields under investigation clustered together and PERMANOVA indicated a significant effect of field on the composition of the rhizobiome (*p* < 0.001, $R^{2}=0.20$) (Figure 3A). Field‐specific community comparisons revealed the presence of distinct fungal communities in the putatively conducive and suppressive sub‐patches of each field (see PERMANOVA results, Figure 3B–D). For all fields, the variable ‘sub‐patch’ (i.e., putatively conducive or suppressive) explained a substantial part of the variation in fungal communities ($R^{2}>20\%$).

Also the composition of the bacterial communities was significantly different between the four field locations (*p* < 0.001, PERMANOVA) (Figure 4A). Analysis of individual fields showed a clear separation in community composition between putatively suppressive and conducive soil in fields E and G (Figure 4B,D, respectively) (PERMANOVA, *p* < 0.05). The bacterial community composition in fields K and S (Figure 4C,E, respectively) was not significantly affected by the variable sub‐patch. PCoA plots and PERMANOVA analyses were also generated using the Aitchison distance and this produced similar results for the fungal and bacterial communities (respectively, Figures S3 and S4) except for the fungal communities in fields K and S. These results show that the fungal and bacterial rhizosphere communities from potato plants grown in putatively suppressive and conducive soil for 13 weeks differ in composition.

### 3.3 Intra‐ and Interfield Composition of Nematode Antagonist Communities

**FIGURE 5:**
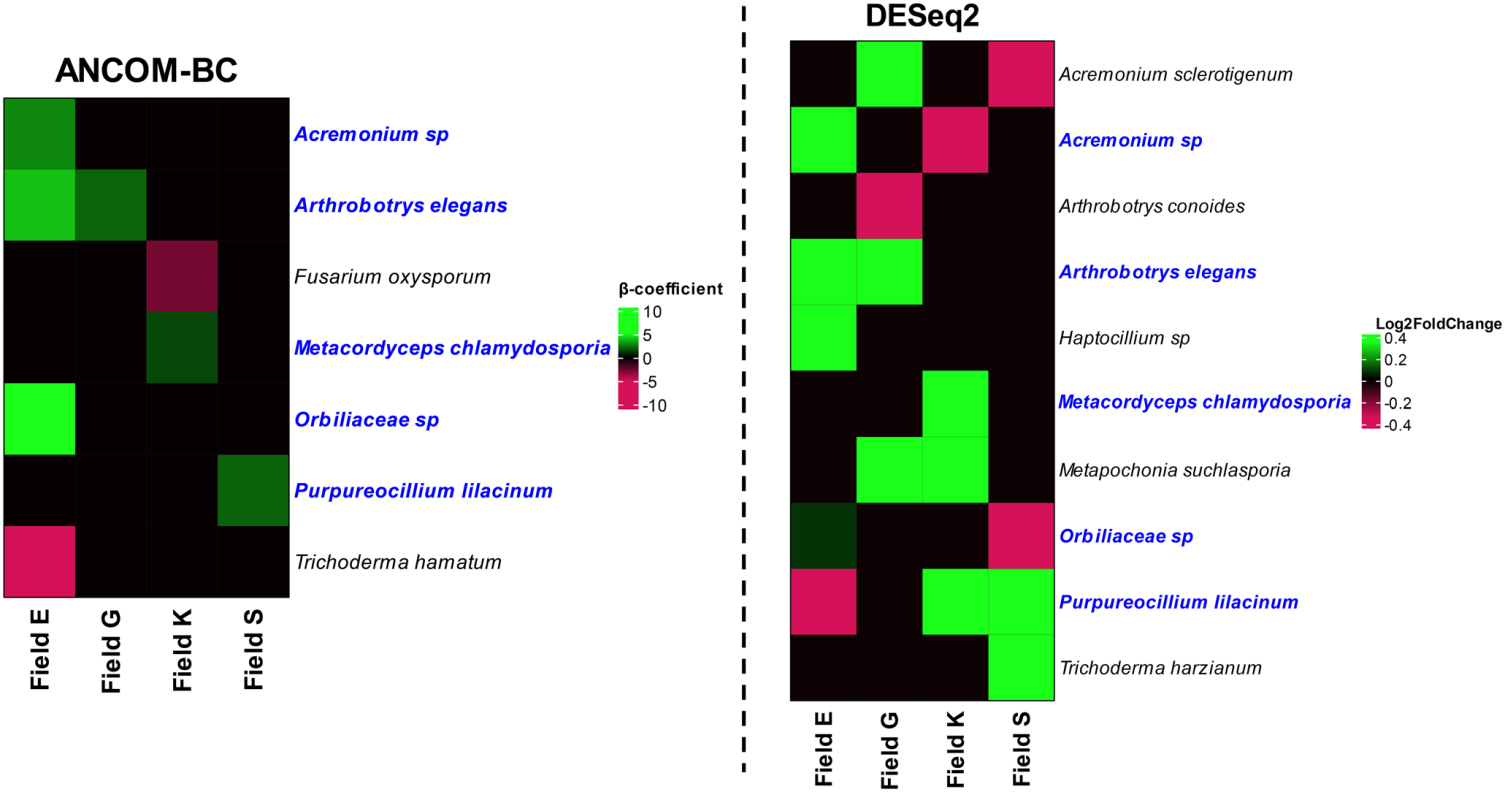
Differentially abundant (putative) fungal nematode antagonists as revealed by ANCOM‐BC (left) or DESeq2 (right) (adjusted *p* value < 0.05). Taxa with a positive *β*‐coefficient or log2 fold change (= green) were more abundant in the putatively suppressive sub‐patches, while taxa with a negative *β*‐coefficient or log2 fold change (= red) were less abundant in the putatively suppressive sub‐patches. Taxa indicated in blue were differentially abundant in both analyses.

Two distinct algorithms, ANCOM‐BC and DESeq2, were used to determine which fungal and bacterial taxa are differentially abundant in the suppressive and conducive sub‐patch of each field. When data from all field locations were aggregated, ANCOM‐BC revealed 1 fungal taxon and DESeq2 17 fungal taxa that were differentially abundant between the two distinct sub‐patches. The latter 17 taxa included the nematode antagonists *Arthrobotrys elegans* (more abundant in putatively suppressive sub‐patch) and *Trichoderma hamatum* (less abundant in putatively suppressive sub‐patch). Differential abundance analyses at field level revealed a higher abundance of fungal taxa known to interact with plants in some of the putatively suppressive sub‐patches. For example, the arbuscular mycorrhizal fungus *Claroideoglomus claroideum* and the endophyte *Coniochaeta* sp. were more abundant in the putatively suppressive sub‐patch of field E (Figure S5). Various fungal nematode antagonists were differentially abundant in the putatively suppressive sub‐patches of the fields (Figure 5). ANCOM‐BC (Figure 5A) analysis revealed that in field E, *Arthrobotrys elegans* (syn: *Orbilia elegans*, *A. oudemansii*; Scholler et al. 2000; Baral et al. 2020), *Acremonium* sp., and an Orbiliaceae species were more abundant in the putatively suppressive sub‐patch. In field G, *Arthrobotrys elegans* was also more abundant. In field K, *Metacordyceps chlamydosporia* (synonymous to *Pochonia chlamydosporia*) was more abundant, while an increase in presence of *Purpureocilium lilacinum* was observed in field S. These results were corroborated by DESeq2 (Figure 5B). Additionally, DESeq2 identified several other nematode antagonists in each field that were more abundant in the putatively suppressive sub‐patches of the fields, for example, *Metapochonia suchlasporia* (field G & K) and *Trichoderma harzianum* (Field S). Both analyses also identified fungal antagonists that were less abundant in the putatively suppressive sub‐patches in each field, for example, *T. hamatum* (field E) and *A. conoides* (field G). Furthermore, some of the nematode antagonists showed inter‐field differences in differential abundance between the two sub‐patches. For example, *P. lilacinum* was more abundant in the putatively suppressive sub‐patches of field K and S, while this fungi was less abundant in this sub‐patch of field E (DESeq2). These results show that various fungal nematode antagonists are associated with the putatively suppressive sub‐patches of each field and suggest that this is field specific.

**FIGURE 6:**
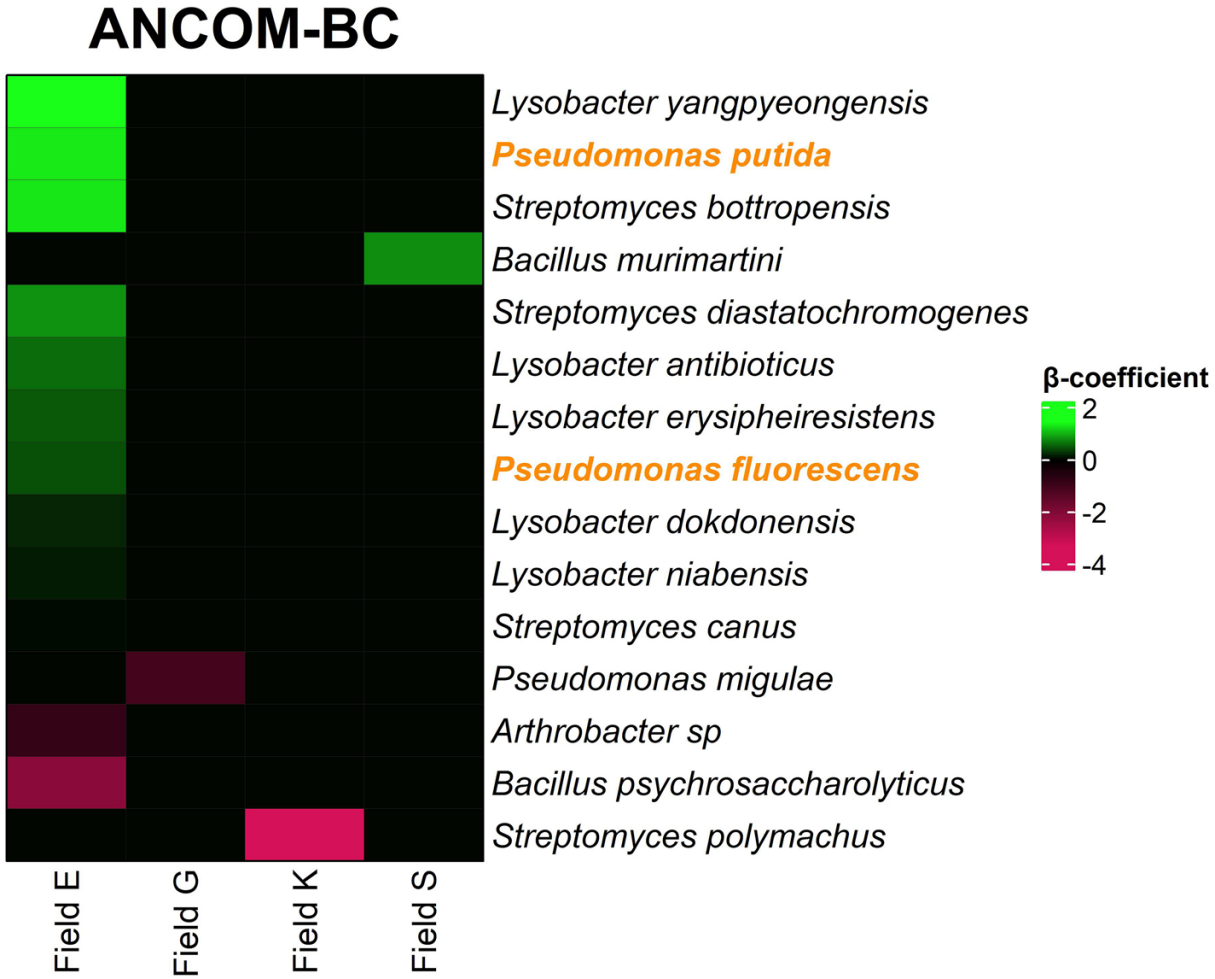
Differentially abundant putative and potential bacterial nematode antagonists as revealed by ANCOM‐BC (Holm adjusted *p* value < 0.05). Bacterial species for which nematode‐antagonistic strains have been described are highlighted in orange. The other taxa (black) represent bacterial genera that harbour nematode antagonists, although no relevant information is available for the specific species. Such taxa are labelled as ‘potential antagonists’. Taxa with a positive *β*‐coefficient (= green) were more abundant in the putatively suppressive sub‐patches, while taxa with a negative *β*‐coefficient fold change (= red) were less abundant in the putatively suppressive sub‐patches.

When data from all field locations were aggregated, ANCOM‐BC revealed 6 and DESeq2 10 bacterial taxa that were differentially abundant between the two distinct sub‐patches. These differentially abundant taxa did not include known nematode antagonists. At field level, ANCOM‐BC analyses identified differentially abundant bacterial taxa (Figure 6) that are known as nematode antagonists and representatives of bacterial genera that are known to comprise antagonists (e.g., Topalović et al. 2020; Li et al. 2015; Stirling 2014). Although these bacterial genera comprise nematode antagonists, no information is available about the trophic ecology of some of these species and therefore they are labelled as ‘potential nematode antagonists’. Nearly complete 16S rRNA sequences allowed us to identify nearly all potential bacterial antagonists at species‐level. Two of the differentially abundant bacterial species are known nematode antagonists *Pseudomonas fluorescens* and *P. putida* , and these species were more abundant in the putatively suppressive sub‐patches of field E. The largest other contrasts were found in field E, where several species of *Lysobacter*, and *Streptomyces* were more abundant in the putatively suppressive sub‐patch, while an *Arthrobacter* and *Bacillus* species were underrepresented. In field G and field K two potential nematode antagonists, respectively *Pseudomonas migulae* and *Streptomyces polymachus*, were less abundant in the putatively suppressive sub‐patches. In field S, *Bacillus murimartini* was shown to be more abundant in the putatively suppressive sub‐patch of the field. These results show that—except of field E—the diversity of (potential) bacterial antagonists that are associated with the putatively suppressive soils is relatively low.

### 3.4 Abiotic Description of the Fields

Due to the limited number of samples (*n* = 2) analysed for chemical analysis, these data are described but not statistically analysed. Chemical analysis of two samples of each sub‐patch of each field revealed no obvious differences in total N, organic matter, organic carbon, and moisture (Figure S6). Notably, a pH difference was observed between the sub‐patches of field E, and one sample of the putative conducive sub‐patch of soil S showed a relatively high total P value.

## 4 Discussion

Natural suppression of soil‐borne plant pathogens by microbiota is an interesting and biologically complex phenomenon. Comparison of microbial communities from conducive and pathogen‐suppressive fields as a means to pinpoint this phenomenon seems straightforward, but relevant biological signals could be obscured by variability in field conditions unrelated to disease suppressiveness. Due to their quarantine status, potato cyst nematodes (PCN) are extensively monitored in potato production areas and, depending on local phytosanitary legislation, within‐field PCN distribution data might be available. Among distribution data from numerous fields, we selected four fields that showed patchy PCN distributions that could not be attributed to obvious local field variables. Upon testing PCN reproduction on soils from putatively conducive and suppressive field patches under greenhouse conditions, local PCN suppressiveness as well as its biological origin were confirmed. Characterisation of microbial rhizosphere communities revealed nematode antagonists positively associated with this local PCN suppression. Notably, the conglomerate of antagonists associated with PCN suppression appeared to be specific for each of the fields, and different among sub‐patches within each field.

### 4.1 The Biotic Origin of Local PCN Suppression

The γ‐irradiation‐based inactivation of the soil microbial community was used to verify the biotic origin of PCN suppression. This verification approach has previously also been used to show the biotic origin of suppression of the fungus *Fusarium culmorum* on wheat (Ossowicki et al. 2020), and of the root‐knot nematode *Meloidogyne javanica* on cucumber (Watson et al. 2020). In all the putative suppressive patches of the fields, no PCN was detected. However, although reproduction was reduced, PCN was still able to multiply in the greenhouse in pots with 30% suppressive soil in which fully susceptible potato was grown. Evidently, greenhouse conditions were different from the field conditions especially regarding soil temperature and moisture content. As a consequence, the PCN suppressive nature of these soils might have decreased under altered environmental conditions. Comparison of the sterilised and non‐sterilised soil of the conducive sub‐patch also showed an increase of the number of PCN cysts in the conducive soil upon sterilisation. However, the percentage of increase and effect size as determined by the ‘estimate’ was substantially lower as compared with the suppressive soils. We hypothesise that sterilisation of the soil eliminated any biological competitors of the inoculated PCN. Thus, the dominant microbial community in the untreated conducive soil contributed to a mild general suppression effect (Weller et al. 2022).

Suppressiveness could result from a reduced number of cysts formed, but also from a reduction in the number of viable content per cyst (Eberlein et al. 2016; Indarti et al. 2021; Nagachandrabose 2020). In our study, the overall number of cysts was reduced in the putatively suppressive soil, but the overall viable content of the cysts (i.e., number of living larvae) was not significantly affected. This suggests that due to biotic factors, fewer juveniles could reach and/or successfully penetrate the roots of a suitable host (i.e., fewer cysts). Once infective 2nd‐stage juveniles had established a feeding site, the soil microbiome from PCN suppressive field appeared to have no effect on fertilisation and subsequent maturation of fertilised eggs. Alternatively, some biotic factors might have inhibited proper female maturation, which also could result in fewer extracted cysts.

### 4.2 Nematode Antagonists Associated With Suppressive Field Sub‐Patches

Characterisation and comparison of bacterial and fungal communities in putatively conducive and suppressive sub‐patches of the fields revealed various fungal and bacterial nematode‐antagonistic taxa that were more abundant in the putatively suppressive sub‐patches. Outside of the center of PCN diversity (Andean region in South America), PCN suppressive soils are thought to be uncommon (Kerry et al. 2009). In contrast to *Meloidogyne* suppressive soils (e.g., Watson et al. 2020; Adam et al. 2014; Bell et al. 2016), little is known about local soil microbiomes that could suppress PCN. Nevertheless, multiple bacterial and fungal isolates from soils originating from Ireland (Cronin et al. 1997), Portugal (dos Santos et al. 2013), Tunisia (Hajji et al. 2017), and India (Nagachandrabose 2020) have been identified as PCN antagonists. Furthermore, a recent survey identified several (abundant) taxa in a PCN suppressive soil from Scotland (UK) (PhD thesis Jones 2024), including those that were abundant in the putatively suppressive sub‐patches in our study (e.g., *Arthrobotrys* sp. and *Streptomyces* sp.).

Several known PPN antagonists were more abundant in the putatively suppressive soils included in our study, such as *Purpureocillium lilacinum*, *Arthrobotrys elegans*, *Metacordyceps chlamydosporia*, and *Pseudomonas fluorescence*. The potential of the nematode‐egg parasite *P. lilacinum* for the control of PPNs—including PCN—has been well documented (e.g., Hajji et al. 2017; Singh et al. 2013; Nagachandrabose 2020). Accordingly, several microbial bio‐formulations containing *P. lilacinum* are used as bionematicides in PCN management (Mhatre et al. 2022). The capacity to form nematode‐trapping adhesive networks is conserved across the genus *Arthrobotrys* (Scholler et al. 1999) and numerous members of *Arthrobotrys* have been identified to trap mobile juveniles of various PPNs (e.g., *M. incognita* ; Soliman et al. 2021; *Pratylenchus zeae*; Sankaranarayanan and Hari 2021; *Xiphnema index*; Wernet and Fischer 2023). *Metacordyceps chlamydosporia* parasitizes PCN eggs (Manzanilla‐López et al. 2017) and *Pseudomonas fluorescence* is able to reduce PPN juvenile mobility (Cronin et al. 1997). Hence, these fungal and bacterial taxa collectively had the capacity to parasitize the inoculated PCN eggs and reduce the number of juveniles that reach the roots, resulting in a reduced number of cysts. Apparently, these microbes did not interfere with the induction or maintenance of PCN‐induced feeding structures (syncytia) as this would have affected the viable content of the cysts. Also, several fungal nematode antagonists were more abundant in the putatively suppressive patches of fields where PCN multiplication was not reduced in the greenhouse assay. The lifestyle (i.e., saprotrophic or nematophagous) that many fungal nematode antagonists exert strongly depends on the local environmental conditions (e.g., Nordbring‐Hertz 2004). Exposure of soil to greenhouse conditions represents a change regarding temperature and moisture content, and this might have affected the lifestyle of individual nematode antagonists. Having said this, the microbial communities derived from the field sub‐patches largely exhibited effects consistent with the suppressive or conducive nature of their respective sub‐patch.

Similarly to PCN, the soybean cyst nematode *Heterodera glycines* has spread from its centre of origin (north‐east China) to nearly all major soybean producing regions in the world (Yu 2011). Also for soybean—soybean cyst nematode disease suppressiveness has been documented outside the centre of diversity. For example, Chen (2007) and Hu et al. (2017) identified *H. glycines* suppressive soils in the United States and identified bacterial (e.g., *Lysobacter* spp.) and fungal (e.g., *Hirsutella rhossiliensis*) taxa associated with this suppressive capacity. These studies corroborate our findings that local microbial communities might be able to suppress non‐native soil‐borne pathogens.

Not all nematode‐antagonistic taxa present in putatively suppressive field patches were stimulated upon controlled exposure to PCN‐infected potato plants. Whereas *Pseudomonas* sp. and *Acremonium* sp. were more abundant in the putatively suppressive sub‐patch of field E, the abundance of *Trichoderma hamatum*, an antagonistic fungus able to parasitize PCN eggs (Nyang'au et al. 2023) and *Arthrobacter* spp., a bacterial genus secreting nematode antagonistic volatile organic compounds (Topalović et al. 2020), was suppressed. These results possibly point at competition between PPN antagonists (Thakur and Geisen 2019) and/or differences in feeding strategy of antagonists.

### 4.3 Dissimilarity of Antagonistic Microbial Assemblages Between Geographically Close Fields

Although we acknowledge that the number of fields investigated here is limited, we observed that the composition of the microbial community associated with PCN suppression differed substantially among fields. Contrasts in soil microbiome composition between different agricultural fields in the same geographical area are common (e.g., Castillo et al. 2017), including taxa associated with fields showing distinct levels of (nematode) suppressiveness (Watson et al. 2020; Zhou et al. 2019; Ossowicki et al. 2020). For example, Watson et al. (2020) found a positive correlation of the bacterial order Chthoniobacterales with a particular *Meloidoigyne* suppressive site in Florida (USA), while this same taxon was suppressed in another nearby suppressive site.

The soil microbial community composition is mainly driven by abiotic factors such as pH (Rousk et al. 2010) and organic matter composition (Sun et al. 2016). Therefore, even subtle variation in soil types, soil chemical properties, and soil management (Rousk et al. 2010; Lauber et al. 2008; Harkes et al. 2019) across fields might have co‐shaped the set of antagonists observed in our fields. Apart from soil physical and chemical properties, also ecological processes such as top‐down regulation by microbial predators (Thakur and Geisen 2019) and stochastic soil processes (e.g., priority effects, Powell et al. 2015; Benucci et al. 2023) might have contributed to the shaping of these distinct PCN antagonistic microbial conglomerates in geographically close fields.

## 5 Conclusion

Local within‐field variation in disease suppression could be instrumental in pinpointing the microbial basis of disease suppressive soils. In this respect, the quarantine status of PCN was advantageous in our study, as statutory monitoring obligations resulted in the availability of high‐resolution distribution data. Our findings highlight that the local differences in soil microbiome composition can influence the local establishment of a phytopathogen within a field. While we show the impact of local variation in the soil microbiome, further research should pinpoint the cause of this localised disease suppression. Such insights will advance our understanding of the manipulability and controllability of disease suppressive soils. Distinct conglomerates of antagonists were associated with PCN suppression even in geographically close fields. This finding emphasises the challenges associated with the application of biological control agents to manage PCN and other soil‐borne pathogens. Finally, confirmation of the occurrence of PCN suppressive soils in Europe encourages further efforts to exploit this potential as a new strategy to durably manage this notorious plant pathogen.

## Author Contributions

**Robbert van Himbeeck:** formal analysis, investigation, methodology, visualisation, writing – original draft, writing – review and editing. **Stefan Geisen:** funding acquisition, conceptualisation, methodology, writing – review and editing. **Casper van Schaik:** investigation, writing – review and editing. **Sven van den Elsen:** investigation, writing – review and editing. **Roeland Berendsen:** investigation, writing – review and editing. **André Bertran:** investigation, resources, writing – review and editing. **Egbert Schepel:** investigation, resources, writing – review and editing. **Johannes Helder:** conceptualisation, funding acquisition, methodology, supervision, writing – review and editing.

## Conflicts of Interest

The authors declare no conflicts of interest.

## Supporting information


**Data S1.** Supporting Information.

## Data Availability

Raw demultiplexed 16S nanopore reads and ITS2 Illumina reads are openly accessible in the Sequence Read Archive (SRA) of NCBI under BioProject accession PRJNA1217848.
